# Severe vitamin D deficiency presenting as hypocalcaemic seizures in a black infant at 45.5 degrees south: a case report

**DOI:** 10.1186/1757-1626-1-12

**Published:** 2008-05-29

**Authors:** Katharine Wallis

**Affiliations:** 1 Department of General Practice, Dunedin School of Medicine, University of Otago, P O Box 913, Dunedin, New Zealand

## Abstract

A 5 month old black infant presented to the Emergency Department in the spring with generalised seizures. He was found to have severe vitamin D deficiency and hypocalcaemia. The baby had been born healthy in the autumn at 45.5 degrees south to parents of African extraction, had been fully breast-fed since birth and had spent some time in day care over the winter months. His mother was a professional working woman who had not taken any vitamin or nutritional supplements during pregnancy or lactation.

## Background

Vitamin D deficiency and rickets used to be commonplace however with the discovery that vitamin D was synthesised following sun exposure, and with vitamin D fortification of some common foods, were thought to have been eradicated. Unfortunately, however, vitamin D deficiency and rickets are again becoming a major global health issue [1].

While vitamin D deficiency in children usually presents as rickets, when severe such deficiency may result in hypocalcaemic seizures. Vitamin D deficiency prevents the efficient absorption of dietary calcium and phosphorus leading to an increased secretion of parathyroid hormone which conserves serum calcium by mobilising calcium stores from the skeleton. Serum calcium levels are thus usually preserved in vitamin D deficient children, however when the calcium stores in the skeleton are totally depleted the infant will become hypocalcaemic [1].

## Case Presentation

A 5 month old black infant presented to the Emergency Department in the springtime with generalized seizures. The baby had received his routine 5 month immunisations several hours earlier that day, which he tolerated well, and had no history of health or developmental concerns. The baby had been born healthy at term in the autumn at latitude 45.5 degrees south and had been exclusively breast fed since birth. His parents were professionals of African origin and the baby had spent some time in day care over the winter months. The baby's mother was a fit and well 39 year old lawyer with two older children. She had not taken any nutritional or vitamin supplements during pregnancy or lactation.

On admission the baby was found to have severe vitamin D deficiency, hypocalcaemia and iron deficiency. There were no clinical signs of rickets however wrist X rays revealed evidence of rickets. Urine tests revealed a urinary infection.

Tests results on admission: (see table 1)

**Table 1:** Tests results on admission

25-hydroxyvitamin D	2	nmol/L	(50 – 150)
Calcium	1.38	mmol/L	(2.05 – 2.25)
Corrected calcium	1.25		(2.05 – 2.55)
Alkaline phosphatase	970	U/L	(40 – 130)
Magnesium	0.67	mmol/L	(0.7 – 1.1)

The baby made a full recovery after treatment with calcium, magnesium, vitamin D, iron, and antibiotics.

## Conclusion

Most people, including babies, get most of their vitamin D through exposure to sunlight (UVB) [2] The duration of sun exposure required to produce a given amount of vitamin D depends upon the amount of skin exposed, skin melanin content, use of sun block, glass cover, cloud cover, latitude, season [3] and age. Melanin, sun-block, and glass absorb UVB, diminishing the production of vitamin D from sun exposure by more than 90% [1]. At higher latitudes (above 30 degrees), because of the increased path length for the sun's UVB rays, most UVB is absorbed in the stratospheric ozone meaning that very little vitamin D can be produced in the skin over the winter months [1].

During times of reduced exposure to sunlight, vitamin D status is related to dietary vitamin D intake. It is, however, difficult to achieve adequate vitamin D intake through dietary measures alone and consequently serum vitamin D levels tend to be lower at the end of winter [3] and may become dangerously low in at risk individuals. Breast milk contains very little, if any, vitamin D [4,5] Those most at risk of vitamin D deficiency include pregnant women, breast fed infants and the elderly, in particular those living at high latitudes who have dark skin, wear burka or follow strict sun avoidance practices.

Vitamin D deficiency can cause serious health problems. In addition to rickets and hypocalcaemic seizures, vitamin D deficiency has been implicated in a number of health problems including immune dysfunction, increased risk of type I diabetes [6], increased risk of cancers of the colon, prostate, breast, ovary, oesophagus, and several other tissues [7], as well as falls, poor dental health and cardiovascular disease [8].

To prevent vitamin D deficiency, many northern hemisphere countries have routine vitamin D fortification of foods [9] however in New Zealand we have limited vitamin D fortification, making routine screening and treatment of pregnant women, and routine supplementation of breast fed infants of dark skinned or veiled women, important considerations [10].

## Consent

Written informed consent was obtained from the patient's parents for publication of this case report. A copy of the written consent is available for review by the Editor-in-Chief of this journal.

## Competing interests

The author declares that they have no competing interests.
